# Virulence factors of uropathogenic *Escherichia coli* (UPEC) and correlation with antimicrobial resistance

**DOI:** 10.1186/s12866-019-1587-3

**Published:** 2019-09-02

**Authors:** Chhaya Shah, Ratna Baral, Bijay Bartaula, Lok Bahadur Shrestha

**Affiliations:** 1 Department of Microbiology and Infectious Diseases, B. P. Koirala Institute of Health Sciences (BPKIHS), Sunsari, Dharan, 56700 Nepal; 2 Department of Internal Medicine, B. P. Koirala Institute of Health Sciences (BPKIHS), Sunsari, Dharan, 56700 Nepal

**Keywords:** UPEC, MDR, Virulence factor, Hemagglutinin, Hemolysin

## Abstract

**Background:**

*Escherichia coli* has found to be the predominant uropathogen (50–90%) in uncomplicated, community acquired urinary tract infection (UTI). Uropathogenic *Escherichia coli* (UPEC) express a multitude of virulence factors, which enable the bacteria to establish UTI. The objective of this study was to evaluate the presence of different phenotypic virulence markers in UPEC isolates and determine their correlation with antibiotic resistance pattern.

**Results:**

Out of 105 patients, 56 (53%) were females and 49 (47%) were males. The age of the patients in the study ranged from 18 years to 87 years and majority of the patients belonged to the age group 20–29 years. Virulence factor was observed in 65% (n = 69) of UPEC and 20% (n = 22) of control isolates (P = 0.0001). Haemolysin production was observed in 34(32.3%) of uroisolates and 12 (11.4%) of control strain. Similarly, 62% of UPEC and 1% of control produced biofilm (P = 0.0001). The expression of Mannose-resistant hemagglutinin (MRHA) and mannose-sensitive hemagglutinin (MSHA) in uroisolates were 52.3% (n = 55) and 5.7% (n = 6) respectively, whereas in faecal isolates, 8.5% (n = 9) expressed MRHA and none produced MSHA. Antimicrobial resistance showed a high degree of resistance towards ampicillin, cotrimoxazole and norfloxacin. The resistance was observed in significant higher degree in biofilm formers as compared to non-formers. MDR and ESBL was observed in 51 and 46% of test strains and 9 and 7.6% of control strains (P = 0.0001).

**Conclusion:**

A significant association between virulence factors of UPEC and antimicrobial resistance in UPEC was present. Routine testing of these factors and co-relation with AMR is recommended. These findings will certainly help understand the pathogenicity and proper management of UTI patients, thus decreasing the improper use of antibiotics.

## Background

Urinary tract infection (UTI) is the most commonly diagnosed urological and renal disease. It is frequently associated with morbidity in both hospitalized as well as outpatient [1]. In 50–90% of all the uncomplicated urinary tract infections, uropathogenic *Escherichia coli* (UPEC) is most common organism seen [2, 3]. UPEC are strains of *Escherichia coli* that divert from their commensal status as intestinal flora, grow and persist in the urinary tract and exhibit diverse array of virulence factors and strategies, which allow them to infect and cause diseases in the urinary tract. These strains of *E. coli* are consistently associated with uropathogenicity and are called as UPEC [4].

The important virulence factors of *Escherichia coli* can broadly be divided into two groups: bacterial cell surface and secreted virulence factor. Bacterial cell surface virulence factors most commonly include fimbriae like mainly type 1 fimbriae and P fimbriae. These fimbriae help in adhesion to host cell surface, tissue invasion (which is important in pathogenesis of UPEC causing UTIs), biofilm formation and cytokine induction. Bacterial cell surface virulence factor also include flagellum, capsular lipopolysaccharide and outer membrane proteins. Haemolysin and siderophores are secreted virulence factor. [5, 6]. These virulence factors are important in enabling the bacteria to colonize the urinary tract and persist despite the effectively functioning host defense mechanism [7].

The emergence of drug resistant microorganism among UPEC strains increase the serious threat to global health [8]. Therefore, knowledge regarding local prevalence of UPEC and antimicrobial resistance is essential for optimal management of UTI. The study was thus designed with an objective to evaluate the presence of different phenotypic virulence markers in UPEC isolates and determine their correlation with antibiotic resistance pattern.

## Results

During the study period, 1142 urine samples were obtained from internal medicine ward from same number of patients; 184 yielded bacterial growth with an infection rate of 16.11%. Among them, 105 (57%) were *Escherichia coli*. Out of 105 patients with UTI due to *E. coli*, 56 (53%) were females and 49 (47%) were males. The age of the patients in the study ranged from 18 years to 87 years. Majority of the patients belonged to the age group 20–29 years (36, 34.28%) and 30–39 years (36, 34.28%). One or more virulence factor was expressed by 65% (n = 69) test strains and 6% (n = 6) control strains. Hemolysin production was observed in 32.3% (n = 34) of uroisolates and 11.4% (n = 12)) of control strain, which was statistically significant (P = 0.002). Biofilm production was noted in 62% (n = 65) of UPEC while only 1% (n = 1) control isolate produced biofilm (P < 0.0001). Similarly, MRHA and MSHA were identified in 55 (52.3%) and 6 (5.7%) respectively in UPEC, while commensal *E. coli* showed 8.5% (n = 9) MRHA and no MSHA. Gelatinase production was detected in none of the isolates (Table 1).

**Table 1:** Virulence marker of UPEC and control

	Virulence markers	UPEC (105)	Control (105)	P value	Remarks
1	Hemolysin production	34 (32.3%)	12 (11.4%)	P = 0.002	Significant
2.	Biofilm production	65 (62%)	1 (1%)	P = 0.0001	Significant
3.	Hemagglutinin			P < 0.0001	Significant
	MRHA	55 (52.3%)	9 (8.5%)		
	MSHA	6 (5.7%)	0 (0%)		
4.	Gelatinase	0	0	NA	

NA: Not applicable

Antimicrobial resistance was compared between the test strains and the commensal isolates. The test strains shows significantly higher degree of resistance as compared to the control strains (Table 2). Amikacin was resistant to 22% of UPEC, while only 10% resistance was noted in control strains (P = 0.01). Norfloxacin resistance was noted in 46% of isolates and 15% of control strains (P = 0.0001).

**Table 2:** Comparison of antibiotic resistance (%) between UPEC and commensal isolates

Antibiotic	UPEC (n = 105)	Commensals (n = 105)	P value
Amikacin	22	10	0.01
Ampicillin	51	30	0.002
Ceftriaxone	40	20	0.0016
Cotrimoxazole	46	15	0.0001
Meropenem	20	2	0.0001
Nitrofurantoin	28	12	0.0038
Norfloxacin	43	15	0.0001

The correlation between the virulence factors along with the antimicrobial resistance was also analyzed (Table 3). The hemolysin producing strains showed a higher degree of resistance as compared to non-producing ones. Similar pattern of resistance was also observed among biofilm producing and non-producing strains. The level of resistance to amikacin (30% vs 7.5%, p value = 0.005), ampicillin (67% vs 25%, P = 0.0001), ceftriaxone (49% vs 27%, p value = 0.028), meropenem (26% vs 10%, P = 0.044), nitrofurantoin (38% vs 12.5%, P = 0.04) and norfloxacin (52% vs 30%, P = 0.041) was higher in biofilm formers than non-formers. Moreover, there was significant association found between the hemagglutinin positivity and resistance to antimicrobial agents.

**Table 3:** Relationship between antimicrobial resistance and virulence factor genes in UPEC

	Antibiotic resistance (%)						
Virulence marker	Amikacin	Ampicillin	Ceftriaxone	Cotrimoxazole	Meropenem	Nitrofurantoin	Norfloxacin
Hemolysin							
Positive (n = 34)	41	88	73	85	35	50	70
Negative (n = 71)	12	33	25	28	12	18	30
P value	0.001	0.0001	0.0001	0.0001	0.007	0.001	0.003
Biofilm							
Positive (n = 65)	30	67	49	60	26	38	52
Negative (n = 40)	7.5	25	27	25	10	12.5	30
P value	0.005	0.0001	0.028	0.375	0.044	0.04	0.041
Hemagglutinin							
MRHA positive (n = 55)	32	69	52	67	23	36	58
MRHA negative (n = 50)	10	32	28	24	16	20	28
P value	0.005	0.001	0.01	0.126	0.329	0.064	0.012
MSHA positive (n = 6)	66	83	83	83	50	83	83
MSHA negative (n = 99)	19	49	38	44	18	25	41
P value	0.006	0.088	0.030	0.001	0.05	0.002	0.179

Prevalence of MDR was found to be much higher in case of UPEC isolates as compared to the test strains (51% vs 9%, P = 0.0001). Similarly, comparison of MDR between virulence positive UPEC and virulence negative UPEC also showed significant differences (69% vs 16%, P = 0.0001). The comparative study of ESBL also showed similar result (Table 4).

**Table 4:** Comparison of MDR and ESBL between test and control, virulence positive and negative UPEC

	UPEC (n = 105)	Commensal (n = 105)	P value	Virulence Positive UPEC (n = 69)	Virulence negative UPEC (n = 36)	P value
MDR	51% (n = 54)	9% (n = 10)	0.0001	69% (n = 48)	16% (n = 6)	0.0001
ESBL	46% (n = 49)	7.6% (n = 8)	0.0001	63% (n = 44)	13% (n = 5)	0.0001

## Discussion

Characterization of UPEC isolates and their correlation with antibiotic resistance patterns in patients with UTI are not well known, particularly in Nepal. Understanding of virulence factors certainly aids in the proper management of UTI and eventually, the prevention of antimicrobial resistance.

In our study, UTI was more prevalent in females, with the total of 105 cases which constitutes 53%. The result is in agreement with several other studies [9, 10]. The incidence of UTI was more in the age group 20 to 39 with age group of the patient ranging from 17 years to 87 years. This result was supported by previous study of Hooton et al. [11], Kabugo et al. [12] and Jadhav et al. [13]. The possible explanation of this may be because most of these patients were female and women of the reproductive age group, who are most vulnerable, due to short urethra, or close proximity of the urethra to anal canal and vagina [4, 14].

Our study showed greater production of hemolysin by UPEC (32.3%, n = 34) than the control strains (11.4%, n = 12), which is statistically significant (P < 0.0001). This finding is consistent with several studies done previously [13, 15, 16]. A similar study done by Mittal et al. [9] revealed a higher rate of hemolysin production(47%) in UPEC, while Kauser et al. [17] observed only 21% were hemolysin producer. The toxin, hemolysin, is a vital virulence factor of UPEC which targets multiple host pathways to facilitate infection [18].

In the present study, biofilm formation was evident in majority of UPEC; this is in agreement with study done by Karam et al. [15], Sharma et al. [19] and Dumaru et al. [20]. Study done by Fattahi et al. [21] have observed a higher rate of biofilm formation (90%). Soto et al. [22] and Karam et al. [15] concluded that biofilm formation was higher in UPEC than in control strains, which is in concordance with our finding. Biofilm is one of the important virulence factors of UPEC protect them from host immunity and antimicrobial agents causing persistent infections [22].

We observed a notable difference in MRHA expression in test isolates as compared to the control (52.3% vs 8.5%, P = 0.0001); the results are consistent with study done by Raksha et al. [2], Najar et al. [23] and Vagarali et al. [7]. The type 1 fimbriae, MSHA was expressed in 5.7% of UPEC and none of the commensal strains, as per our research. Kaira et al. [24] demonstrated expression of 41% MRHA and 5% MSHA in their study; the results are in agreement with our finding. Fimbriae (P fimbriae and type 1 fimbriae) plays an important role in pathogenesis of UTI by facilitating the attachment of *E. coli* to the uroepithelium [7].

None of the isolates in our study produced gelatin, which was in accordance to study by Kaira et al. where none of the isolates showed gelatinase production and EL-Mosallamy et al. where only 1(2%) showed gelatinase production [24, 25]. However, another study has demonstrated a high degree of gelatin production [9]. Gelatinases is an important virulence factor of *E. coli* responsible for pathogenicity in different diseases, particularly UTI [2, 5].

The study of antimicrobial resistance showed a variable degree of resistance among UPEC isolates; 51% were resistant to ampicillin, 43% to norfloxacin and 20% to Meropenem. The comparison of antimicrobial resistance among UPEC and control strains showed a significant difference. The results agree with the study done by Karam et al. [15]. and Li et al. [26]. Multi-drug resistance was observed at a higher rate among UPEC strains as compared to control (51% vs 9%, P = 0.0001). Similarly, ESBL production was significantly higher in test strains in comparison with the commensals (46% vs 7.6%). The findings are similar to study conducted by Tabasi et al. [8] and Karam et al. [15]. Other studies have shown similar prevalence of ESBL production among UPEC [27, 28]. Jadhav et al. (21.3%) have reported a lower incidence of ESBL and Mittal et al. (29.62%) have reported lower incidence of MDR [9, 13]. The higher rate of MDR and ESBL in our setting might be due to the easy availability of antimicrobial as over the counter medication in our country.

Antimicrobial resistance was significantly higher in biofilm producing UPEC as compared to biofilm non-producers. The finding is in concordance with studies done by Karam et al. [15], Tadepalli et al. [29] and Tabasi et al. [8]. Several studies done in the past also conclude the same findings [30, 31]. Pompilio et al. [32] concluded that, although antimicrobial resistance was higher in biofilm producers, the difference was statistically non-significant. Protection of microorganism from host immunity, insufficient antimicrobial concentration and delayed penetration into the deeper layers of biofilms are the major reasons for antibiotic resistance in biofilm structures [22].

The study emulated a significant correlation between the hemolysin production and antimicrobial resistance. Amikacin was resistant in 41% of hemolysin positive strains while only 12% resistance was observed in hemolysin negative strains (P = 0.0001). Similar pattern of resistance was observed against other antimicrobial agents. Similarly, the UPEC isolates demonstrating P fimbriae (MRHA) and Type 1 fimbriae (MSHA) showed higher degree of resistance than the ones without these virulence factors. The findings are similar to studies conducted in the past [8, 15]. Fimbriae (Type 1 fimbriae and P fimbriae) help in adhesion, quorum sensing and biofilm development which directly or indirectly contributes to the development of resistance to antimicrobials [33].

The prevalence of MDR (69% vs 16%, P = 0.0001) and ESBL (63% vs 13%, P = 0.0001) was markedly higher in virulent UPEC as compared to non-virulent UPEC strains. The findings are similar to study done by Jadhav et al. [13], Karam et al. [15] and Tabasi et al. [8].

## Conclusion

We found a very significant association between virulence factors of UPEC and antimicrobial resistance in UPEC. Routine testing of these factors and co-relation with AMR is recommended. These findings will certainly help understand the pathogenicity and proper management of UTI patients, thus decreasing the improper use of antibiotics.

## Methods

This is a prospective study, which was conducted in a period of one year from September 2017 to August 2018 in the department of Microbiology and infectious diseases in collaboration with department of Internal medicine at B.P. Koirala Institute of health sciences (BPKIHS) Dharan, Nepal. Urine samples were obtained from patients from Internal medicine unit who were symptomatic for UTI. A total of 105 isolates of *Escherichia coli* were taken from the urine and the same number of control were used (faecal isolates from apparently healthy individuals). The isolates were identified by standard microbiological methods.

### Detection of hemolysin production [2, 8, 34]

The organism from the bacterial stock vial was inoculated into peptone water till turbidity matching 0.5 Mc Farland was obtained. Thereafter the organism was inoculated onto 5% sheep blood agar and incubated overnight at 35 °C. Hemolysin production was detected by complete hemolysis or Beta hemolysis of the erythrocytes on blood agar indicated by clearing around the colony. Plate was also kept at 4 degree centigrade overnight to clearly observe the hemolysis.

### Biofilm production [35]

Biofilm production was detected by using congo red gar method proposed by Freeman et al. [35]. Congo red agar was prepared by mixing brain heart infusion broth, sucrose, congo red dye and agar (HiMedia) in 1 l distilled water. The organisms were plated on it and incubated aerobically at 37 °C for 24 h. The observation of black colored colony was considered as biofilm positive and red colored colony as negative.

### Hemagglutination test [9, 34, 36]

The test was performed according to the direct bacterial hemagglutination test-slide method. Human blood group ‘O’ obtained from the blood transfusion service was centrifuged and washed thrice in 0.85% (w/v) NaCl solution (saline) and made into 3% RBC suspension. Colonies from overnight growth of *E. coli* from the plate, were inoculated into 1% nutrient broth. It was then incubated for 48 h till full fimbriation. It was centrifuged and the bacillary deposits obtained were re-suspended in a small amount of residual fluid.

One drop of 3% RBC suspension was added to a drop of the bacillary culture. The tile was rocked at room temperature by tilting it to and for 10 min. If clumping was positive, it was said to be positive for hemagglutination. In a parallel test, a drop of 2% (w/v) D-mannose was added to bacillary culture and 3% RBC for which absence of hemagglutination was taken as MSHA) and presence of hemagglutination as MRHA.

### Gelatinase test [9, 34]

The test was done by gelatin plate method where gelatin nutrient agar was used to test for gelatinase production by the bacteria. The plate was inoculated with the heavy inoculum of an overnight old strain of *Escherichia coli*. The growth of the organism was observed and thereafter plate was flooded with mercuric chloride solution. The development of opacity in the medium and a zone of clearing around the colonies on flooding with mercuric chloride solution is indicative of gelatin liquefying colony and was considered positive for gelatinase.

### Antimicrobial susceptibility testing [37]

It was performed according to standards of Clinical and laboratory Standards Institute (CLSI) guideline using Kirby Bauer’s disc diffusion method on Muller-Hilton agar (HiMedia laboratories). Antibiotics discs such as amikacin 30 μg, ampicillin 10 μg, ceftriaxone 30 μg, Norfloxacin 10 μg, cotrimoxazole (trimethoprim/ sulfamethoxazole) 1.25 μg/23.75 μg, Meropenem 10 μg, nitrofurantoin 300 μg were tested.

### Statistical analysis

Data were collected and entered into a database using MS Excel 2013. Statistical analysis was conducted using SPSS version 16. Chi-square test was applied for comparison of categorical variables. The strength of the relationship and their 95% of confidence interval was calculated. P value less than 0.05 was considered as statistically significant.

## Data Availability

Yes, available from corresponding author upon request.

## Abbreviations

ESBL: Extended spectrum β-lactamase
MDR: Multi-drug resistant
MRHA: Mannose-resistant hemagglutinin
MSHA: Mannose-susceptible hemagglutinin
UPEC: Uropathogenic *Escherichia coli*
UTI: Urinary tract infection
